# Investigating the Role of BAFF and Its Receptors in Renal Transplant Recipients with Chronic Antibody-Mediated Rejection

**DOI:** 10.1155/2021/6654992

**Published:** 2021-03-06

**Authors:** Shima Afzali, Saeedeh Salehi, Abbas Shahi, Marzie Esmaeili, Samad Farashi Bonab, Azin Peykari, Farzaneh Bagherpour, Bita Ansaripour, Tayebeh Soleimanian, Fatemeh Pour-Reza-Gholi, Aliakbar Amirzargar

**Affiliations:** ^1^Department of Immunology, School of Medicine, Tehran University of Medical Sciences, Tehran, Iran; ^2^Students' Scientific Research Center, Tehran University of Medical Sciences, Tehran, Iran; ^3^Chronic Kidney Disease Research Center, Labbafinejad Medical Center, Shahid Beheshti University of Medical Sciences, Tehran, Iran; ^4^Organ Procurement Unit, Sina Hospital, Tehran University of Medical Sciences, Tehran, Iran; ^5^Nephrology Research Center, Shariati Hospital, Tehran University of Medical Sciences, Tehran, Iran

## Abstract

**Background:**

Kidney transplantation is the best treatment option for end stage renal disease (ESRD), but graft rejection is still a big obstacle that occurs in spite of immunosuppressive therapy. B cells are considered as the major reason for renal graft rejection because of antibody production. Due to their roles in B cell function, we intended to evaluate the B cell activating factor (BAFF) and its receptors including BAFF receptor (BAFF-R), B cell maturation antigen (BCMA), and transmembrane activator and cyclophilin ligand interactor (TACI) in renal transplant patients.

**Method:**

The study included 40 kidney allograft patients with cAMR, 40 stable kidney allograft patients, and 8 healthy volunteers with normal kidney function. The percentage and absolute number of CD19^+^ B cells were analyzed by flow cytometry, the serum level of BAFF was analyzed by ELISA, and mRNA expressions of BAFF and BAFF receptors (BAFF-R, BCMA, and TACI) were measured using quantitative real-time PCR.

**Results:**

The percentage and the absolute number of B cells decreased significantly in stable and cAMR patients compared to healthy individuals. The serum level and gene expression of BAFF, as well as the mRNA level of BCMA, were increased significantly in both cAMR and stable patients compared to healthy volunteers. There was an overexpression of TACI mRNA in cAMR patients compared to stable patients.

**Conclusions:**

Both soluble protein and mRNA transcript of BAFF increased in transplant recipients. However, BAFF neither at the serum level nor at the mRNA transcript level cannot be a good biomarker for the prediction of cAMR. In addition, expression of TACI, compared to other receptors of BAFF, confers a potential to be used in distinguishing cAMR and stable kidney transplant patients.

## 1. Introduction

According to the statistics provided by the global observatory on donation and transplantation (GODT), more than 85,000 kidney transplantations have been done worldwide up to 2016 [1]; however, many transplanted patients require retransplantation or even die of graft rejection. Thus, seemingly, the prevention of graft rejection is a critical step in improving the outcome of organ transplantation, and nowadays, a variety of immunosuppressive drugs are used to reduce graft rejection. Currently available drug regimens, mostly focus on T cells and lead to the reduction of acute cellular rejections, but they seem to be inefficient in controlling chronic rejections that are mainly caused by antibody-mediated processes [2, 3]. Although an increase of nearly 90% has been observed in 1-year graft survival, and the incidence of 1-year post-transplantation acute rejection decreased by 12.2% [4], chronic antibody-mediated rejection (cAMR) is considered as the main cause for late allograft loss. The cAMR has a poor prognosis, and conventional immunosuppressive treatment can not prevent it. Besides, the development of cAMR is not fully understood in detail [5].

There are several methods for recognition of antibody-mediated rejection (AMR), include of measuring the serum level of donor-specific antibodies (DSAs) and staining of biopsied tissue for C4d as a complement fragment that has precipitated following antibody activity. However, the serum levels of DSAs and C4d depositions on the graft are not ideal biomarkers [6]. The serum levels of DSAs are often undetectable in the cAMR patients due to their low levels [7]. Moreover, it has been shown that only 30-40% of DSA positive patients develop AMR [8]. Also, cAMR may occur in the lack of DSA [7]. On the other hand, although the evaluation of biopsy is still a gold standard for diagnosis of rejection, it is an invasive method that is often accompanied by bleeding, arteriovenous fistula formation, infection, and even graft loss and death in rare cases [9]. Additionally, it has been shown that AMR cases, especially cAMR, may be found in patients without any C4d deposition [10, 11]. Thus, more reliable and sensitive biomarkers are needed for recognition of cAMR occurrence.

Several mechanisms have been attributed to B cells during graft rejection. The most important function of B cells is producing antibodies against donor human leukocyte antigen (HLA) and non-HLA antigens. These antibodies contribute to complement fixation and cell lysis, antibody-dependent cellular cytotoxicity (ADCC) by NK cells, increasing the thickness of endothelial and smooth muscle cells, activating the platelet, developing inflammatory conditions, thrombosis, and overall reducing of graft function [2, 12]. Moreover, B cells present antigens to T cells and can activate them by providing costimulatory signals through CD28-B7 and CD40L-CD40 interactions. Additionally, B cells produce cytokines, such as interleukin- (IL-) 6 and interferon- (IFN-) *γ*, and contribute to T cell activation. Also, IL-17 that can be generated by B cells stimulates endothelial, epithelial, and fibroblast cells to produce chemokines and cytokines, leading to the recruitment of neutrophils and establishment of inflammatory conditions. Moreover, by producing cytokines like tumor necrosis factor- (TNF-) *α*, B cells directly promote inflammation, endothelial and epithelial cell injuries, as well as inflammatory renal disorders that cause allograft rejection [13]. B cells also involve in the development of lymphoid like structures, named tertiary lymphoid organs (TLOs), at the sites of inflammation that contain both B cells and T cells. In TLOs, B cells undergo affinity maturation, clonal expansion, and class switching, which result in efficient antibody production [14]. Therefore, focusing on B cells during the transplantation can help a better understanding of allograft rejection pathogenesis.

**Figure 1:**
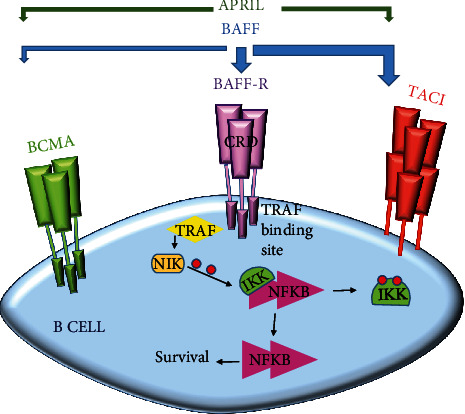
BAFF receptors. BAFF has three receptors on B cells, including BCMA, BAFF-R, and TACI, which usually exist in trimer form on the cell surface. APRIL is another cytokine that acts like BAFF and has two common receptors with BAFF include of BCMA and TACI. BAFF binds to its receptors with different affinity. Thicker arrows show more affinity of BAFF to each receptor. The extracellular domain of receptors consists of cysteine-rich domain (CRD) as ligand binding site (TACI has an extra binding site). Intracellular domains of BCMA and BAFF-R contain a TRAF binding site that initiate the signaling cascade after binding to TRAF, leading to subsequent events like cell survival. BAFF: B cell activating factor; BCMA: B cell maturation antigen; TACI: transmembrane activator and calcium-modulating cyclophilin ligand interactor; APRIL: a proliferation-inducing ligand; TRAF: TNF receptor associated factor; NF-*κ*B: nuclear factor- (NF-) *κ*B; NIK: NF-*κ*B inducing kinase; IKK: inhibitor of *κ*B kinase 1.

B cell-activating factor (BAFF), also known as TNFSF13B, BLyS, TALL-1, and CD257, is a cytokine that belongs to the TNF superfamily members [15]. BAFF is a membrane-bound protein that can undergo proteolytic cleavage in the furin site and convert into a soluble form. Soluble BAFF is generated by antigen-presenting cells (APCs) like B cells, dendritic cells (DCs), monocytes and macrophages, as well as epithelial cells, neutrophils, and activated T cells. BAFF has three receptors on the surface of B cells, including BAFF-receptor (BAFF-R) or BR3, B cell maturation antigen (BCMA), and transmembrane activator and calcium-modulating cyclophilin ligand interactor (TACI). BAFF binds to TACI and BAFF-R with higher affinity in comparison to BCMA (Figure 1) [16, 17]. BAFF receptors have a cysteine-rich domain (CRD) as a ligand-binding site (which is doubled in TACI) and a transmembrane domain, as well as an intracellular domain that contains TNF receptor-associated factor (TRAF) binding site in BAFF-R and BCMA (Figure 1) [15]. The interaction between BAFF and BAFF-R or BCMA activates the noncanonical signaling pathway of nuclear factor- (NF-) *κ*B. Afterward, TRAFs bind to TRAF binding sites and leading to the release of NF-*κ*B inducing kinase (NIK). The inhibitor of *κ*B kinase 1 (IKK1) inhibits NF-*κ*B in a normal situation, but when BAFF stimulates its receptor, the released NIK phosphorylated the IKK1. The phosphorylated IKK1 is removed from NF-*κ*B, and then, NF-*κ*B is activated and moves to the nucleus to carry out its activities. But the TACI signaling pathway is different and is contributed with NF-*κ*B, activator protein (AP)-1, and nuclear factor of activated T-cells (NF-AT). The consequences of the BAFF signaling are included germinal center formation, B cell differentiation and survival, and IgE and IgG class switching, as well as plasma cell survival [17]. Therefore, based on the previous reports on the role of BAFF in activating the B cells and the fundamental role of B cells in cAMR and also due to the few numbers of studies that investigated BAFF and its receptors in cAMR renal transplant patients, we designed this study to evaluate the role of BAFF and its receptors comprise of BAFF-R, BCMA, and TACI in cAMR and stable kidney transplant recipients.

## 2. Material and Methods

### 2.1. Patients

Eighty kidney transplant subjects (53 males and 27 females; with an age range of 18-80 years) were enrolled in this study recruited from a multicenter collaboration of Labbafinejad, Shariati, and Sina hospitals, Tehran, Iran. Of these 80 subjects, 40 patients (with a mean post-transplantation time of 72.55 months) were diagnosed as cAMR through laboratory findings (urea and creatinine levels) and biopsy analysis (C4d deposition and pathological evidence) which have high creatinine concentration (mean: 3.30 mg/dl) and low estimated glomerular filtration rate (eGFR, mean: 49.49 ml/min/1.73 m^2^). Patients with T cell-mediated rejection or cAMR patients that their rejection was not approved by biopsy were excluded from the study. The remaining 40 patients (with a mean post-transplantation time of 69.59 months) had stable graft function without any symptoms of graft rejection, active infection, allergy, and autoimmunity (with mean creatinine concentration: 1.23 mg/dl and mean eGFR: 74.76 ml/min/1.73 m^2^). Additionally, eight healthy volunteers with normal kidney function and no history of kidney diseases were enrolled in the study as the healthy control group (with mean creatinine concentration: 0.81 mg/dl and mean eGFR: 107 ml/min/1.73 m^2^). The patient's demographic data and their baseline characteristics are summarized in Tables 1 and 2. The percentage of lymphocytes was gained from the cell blood count (CBC) of patients at the time of sampling. We calculated the eGFR by Chronic Kidney Disease Epidemiology Collaboration (CKD-EPI) formula shown in Table 3. Patients received two different immunosuppressive protocol; one group received prednisolone, mycophenolate mofetil (MMF), and cyclosporine A (CsA), and the other received prednisolone, MMF, and tacrolimus (Tac) (Table 1). Before sampling, the informed consent forms were obtained from all study subjects. The local ethics committee of the Tehran University of Medical Sciences, Tehran, Iran, approved the protocol of this study (Ethics code No. IR.TUMS.MEDICINE.REC.1396.4308). We collected 5 ml of venous blood for serum isolation, and 10 ml into the ethylenediaminetetraacetic acid (EDTA) anticoagulated collecting tubs for cell isolation and RNA extraction. Blood samples were collected from rejected patients as soon as the rejection was recognized and before initiation of rejection therapy.

### 2.2. PBMC Isolation

Peripheral blood specimens were collected in EDTA sterile tubes, then after that, the ficoll gradient (Inno-train, Germany) was used for isolating the peripheral blood mononuclear cells (PBMCs) from the whole blood. Isolated PBMCs cryopreserved in a media including 10% dimethyl sulfoxide (DMSO, Sigma-Aldrich, UK) and 90% fetal bovine serum (FBS, Thermo Fisher Scientific, Gibco, USA) and then stored in liquid nitrogen tank until use.

### 2.3. Flow Cytometry

**Figure 2:**
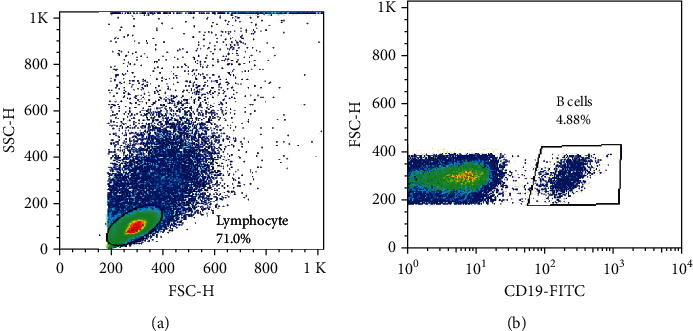
Gating strategy. Forward and side scatter gating for lymphocytes (a). CD19^+^ cells are displayed as B cells in trapezoidal area (b).

For immunophenotyping, the cells gently thawed, and the viability of PBMS was checked by trypan blue dye exclusion method, and PBMCs with more than 90% viability were used for flow cytometry. PBMCs were freshened up in a solution containing RPMI 1640 medium (Biosera, USA) and FBS (10%), and then incubated at 37°C for 15 minutes. Subsequently, phosphate-buffered saline (PBS) and FBS (0.1%) were utilized as a washing buffer to wash out the cells. Then, the cells were suspended in PBS and were stained with anti-human CD19 FITC mAb (clone HIB19, Biolegend, San Diego, CA, USA) according to the manufacture's protocol. CD19^+^ cells were considered as total B cells population (Figure 2). The B cell absolute number was calculated by CBC parameters. The CellQuest software and BD FACSCalibur analyzer were used for cell analysis, and the data analysis was carried out by the FlowJo software 7.6 (Tree Star, Ashland, OR, USA).

### 2.4. Serum Levels of BAFF

The serum of the coagulated whole blood samples was isolated after centrifuging and then stored at -70°C until use. Soluble BAFF levels were measured in the serum samples by enzyme-linked immunosorbent assay (ELISA) following the manufacturer's recommended procedures (R&D Systems, Minneapolis, MN). Values were reported as picograms per milliliter (pg/ml).

### 2.5. RNA Extraction, cDNA Synthesis, and Real-Time PCR

RNA was extracted from the whole blood samples by RNA extraction kit (Thermo Fisher, United States), and RNA quality was determined using the NanoDrop ND-1000 spectrophotometer (Thermo Scientific, USA) by measuring the absorbance at 260/280 and 260/230 nm. Extracted RNAs were reverse transcribed to complementary DNA (cDNA) by reverse transcription system kit (Thermo Fisher, United States). Quantitative real-time PCR was performed using SYBR green master mix and primers (Table 4). Three housekeeping genes, including 18srRNA, *β*-actin, and GAPDH, were checked out, and GAPDH was chosen to normalize the mRNA expression of genes. The alteration in the expression level of each gene was calculated by the comparative Ct method as fold change using the 2^-∆∆Ct^ formula.

### 2.6. Statistical Analysis

The data analysis was performed by the SPSS software (Version 21, Chicago, IL, USA). To determine the normality of scale data distribution, the Kolmogorov-Smirnov test was used. One-way ANOVA was performed to compare the BAFF serum levels between three study groups. *K* independent samples (Kruskal-Wallis *H*) test employed for comparing the number and percentage of CD19^+^ B cells and lymphocytes, as well as mRNA expression levels between three groups. Correlation analysis was conducted to find any relation among the numerical variables. Nonparametric data were shown by the median ± interquartile range (IQR), and parametric data were presented as mean ± standard deviation (SD). *P* values ≤ 0.05 were considered statistically significant.

## 3. Results

### 3.1. Baseline Characteristics

**Figure 3:**
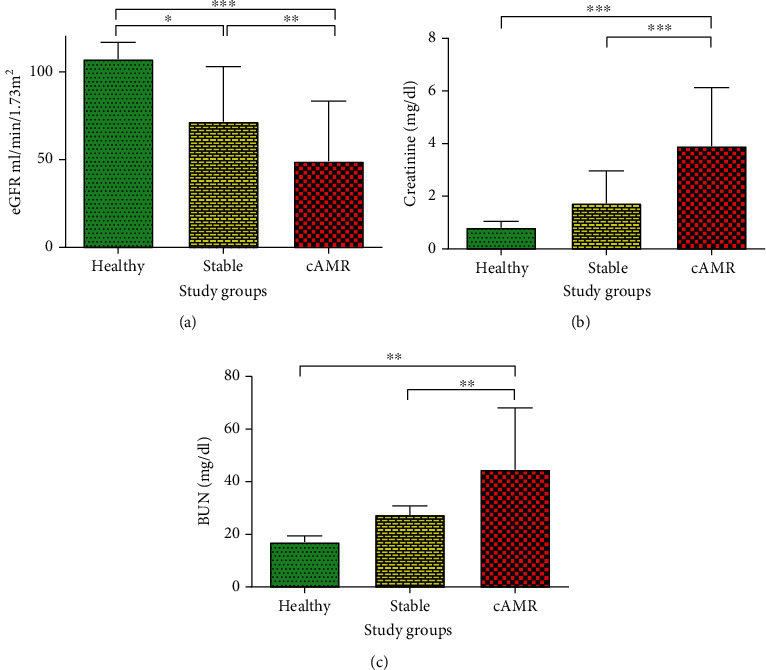
Renal function in the study groups. Estimated glomerular filtration rate (eGFR) (a), serum creatinine concentration (b), and blood urea nitrogen (BUN) (c) which used for evaluating the renal function are compared in healthy individuals, patients with stable graft function, and cAMR patients. Error bars represent SD (^∗^*P* ≤ 0.05, ^∗∗^*P* ≤ 0.01, ^∗∗∗^*P* ≤ 0.001).

Tables 1 and 2 summarize the demographic data and baseline characteristics of enrolled patients and healthy volunteers. Thirteen stable patients received their graft from living, and the remaining 27 stable patients received their graft from deceased donors. Eight cAMR patients received their graft from living donors, and the remaining 32 cAMR patients received their graft from deceased ones (Table 1). eGFR (Figure 3(a)), serum creatinine concentration (Figure 3(b)), and blood urea nitrogen (BUN) (Figure 3(C)) were used for evaluating renal function. cAMR patients had significantly less eGFR, more serum creatinine, and more BUN compared to healthy individuals and stable patients (Figures 3(a)–3(c)).

### 3.2. Percentage and Number of Lymphocytes and CD19^+^ B Cells

**Figure 4:**
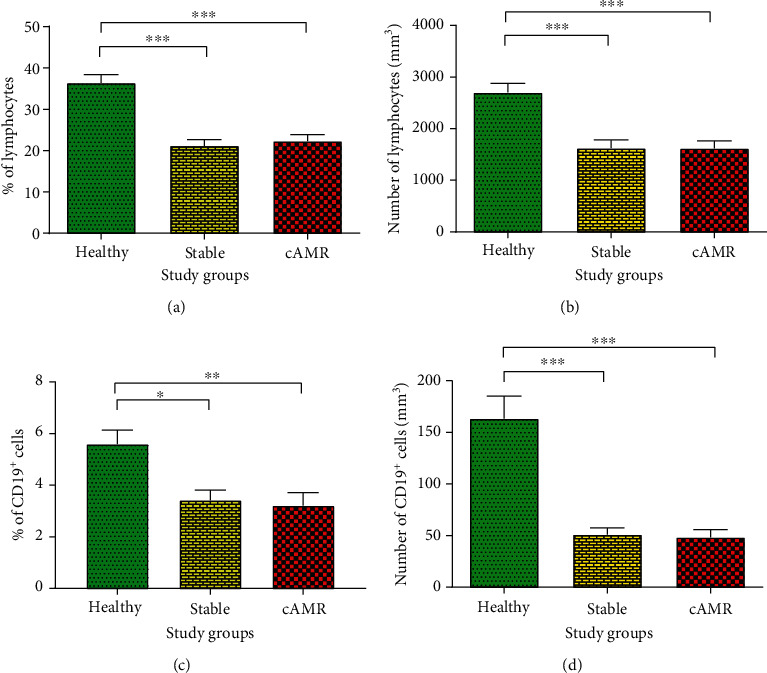
Percentage (a) and absolute number (b) of lymphocytes, as well as percentage (c) and absolute number (d) of CD19^+^ B cells in healthy individuals, patients with stable graft function, and cAMR patients (^∗^*P* ≤ 0.05, ^∗∗^*P* ≤ 0.01, ^∗∗∗^*P* ≤ 0.001).

The percentage of lymphocytes was highly decreased in stable (21.20 ± 9.32%; *P* < 0.001) and cAMR patients (22.32 ± 9.70%; *P* = 0.001) compared to healthy individuals (36.41 ± 5.62%) (Figure 4(a)); however, there was not any significant difference between the lymphocyte percentage of stable and cAMR patients. As well, lymphocyte absolute number had a significant decrease in stable (1600 ± 856.85/mm^3^; *P* = 0.001) and cAMR patients (1517 ± 795.25/mm^3^; *P* = 0.001) in comparison to healthy subjects (2631.5 ± 836.84/mm^3^), while no significant difference was observed between stable and cAMR patients (Figure 4(b)). Also, stable and cAMR patients had a significant decline in their percentage of CD19^+^ B cells (3.30 ± 2.32% and *P* = 0.048 and 2.30 ± 3.00% and *P* =0.006, respectively) compared to healthy individuals (5.77 ± 1.21%) (Figure 4(c)), and also, a significant high decrease in the absolute number of CD19^+^ B cells was observed in stable and cAMR patients (47.95 ± 44.46/mm^3^; *P* = 0.001 and 38.25 ± 45.68/mm^3^;*P* < 0.001, respectively) in comparison to healthy subjects (179.36 ± 80.15/mm^3^), and no significant difference was observed in percentage and absolute number of CD19^+^ B cells between stable and cAMR patients (Figure 4(d)).

### 3.3. Serum Levels of BAFF

**Figure 5:**
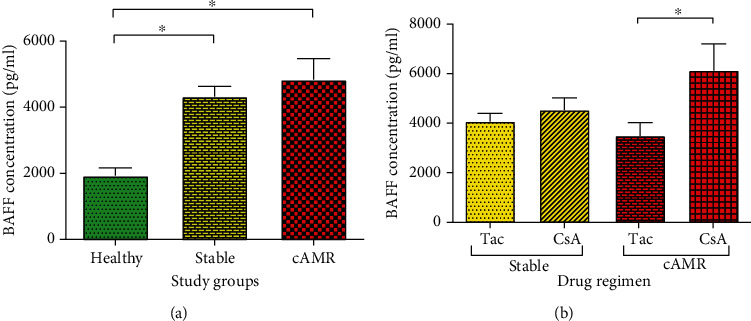
BAFF serum level in healthy individuals, patients with stable graft function, and cAMR patients (a). BAFF serum level in transplanted patients with different immunosuppressive drug, tacrolimus (Tac), or cyclosporine A (CsA) (b). Error bars represent SEM (^∗^*P* ≤ 0.05).

Serum level of BAFF was increased significantly in both stable (4843.9 ± 3906.8 pg/ml; *P* = 0.038) and cAMR patients (4296.1 ± 2008.4 pg/ml; *P* = 0.018) compared to healthy individuals (1931.2 ± 666.7 pg/ml). However, there was not any significant difference between cAMR patients compared to stable group (Figure 5(a)). We classified transplanted patients based on their immunosuppressive drug regimen. The first group received prednisolone, MMF, and CsA (CsA group), and the second group received prednisolone, MMF, and Tac (Tac group). Serum levels of BAFF in cAMR patients who received CsA (6124.5 ± 1074.89 pg/ml) were significantly (*P* = 0.006) higher than cAMR patients in the Tac group (3483.15 ± 540.73, Figure 5(b)). However, there was not any significant difference between CsA stable group and Tac stable group in their soluble BAFF levels. Based on years after transplantation, both cAMR and stable patients were divided into two subgroups, including short-term survival (STS) patients that refer to stable or cAMR patients less than five years post-transplantation and long-term survival (LTS) patients that refer to stable and cAMR patients more than five years post-transplantation. There was no significant difference between STS and LTS stable patients and also between STS and LTS cAMR patients, neither in inter- nor in intragroup analysis (supplementary figure 1).

### 3.4. mRNA Expression of BAFF

**Figure 6:**
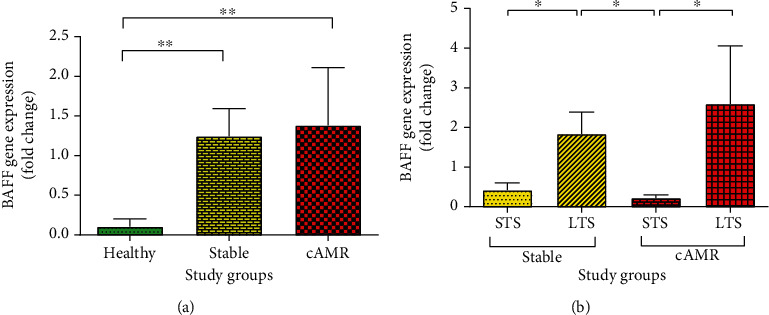
BAFF mRNA expression level in healthy individuals, patients with stable graft function, and cAMR patients (a). BAFF mRNA expression level in stable and cAMR patients with short-term survival (STS) and long-term survival (LTS) (b). Error bars represent SEM (^∗^*P* ≤ 0.05, ^∗∗^*P* ≤ 0.01).

The mRNA expression of BAFF was upregulated in stable (median = 0.25, IQR = 1.25, *P* = 0.002) and cAMR patients (median = 0.23, IQR = 0.90, *P* = 0.004) compared to healthy individuals (median = 0.0005, IQR = 0.01). On the other hand, no statistically significant difference was observed between stable and cAMR patients with respect to the mRNA expression of BAFF (Figure 6(a)). In addition, our results showed that the BAFF mRNA expression in LTS stable patients (median = 0.36, IQR = 3.90) was significantly (*P* = 0.041) higher than STS stable patients (median = 0.08, IQR = 0.22). The same results were seen for cAMR patients; LTS cAMR patients (median = 0.32, IQR = 0.86) showed significant (*P* = 0.039) overexpression of BAFF mRNA compared to STS cAMR patients (median = 0.05, IQR = 0.20) (Figure 6(b)). But no significant difference was obvious in stable and cAMR patients based on their drug regimens (supplementary figure 2).

### 3.5. mRNA Expression of BAFF-R

Our results showed that there was not any significant difference in the BAFF-R mRNA expression level between both cAMR patients and healthy individuals, as well as stable patients and healthy control group. And no statistically significant difference was detected between cAMR and stable patients (supplementary figure 3A). Besides, the division of patients based on the years post-transplantation (supplementary figure 3B), as well as their drug regimen (supplementary figure 3C) did not affect the BAFF-R mRNA expression level, and no significant differences were seen.

### 3.6. mRNA Expression of BCMA

**Figure 7:**
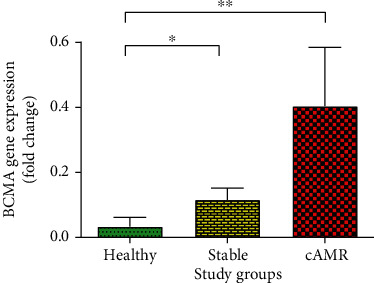
mRNA expression level of BCMA in healthy individuals, patients with stable graft function, and cAMR patients. Error bars represent SEM (^∗^*P* ≤ 0.05, ^∗∗^*P* ≤ 0.01).

Stable (median = 0.02, IQR = 0.10, *P* = 0.02) and cAMR patients (median = 0.07, IQR = 0.18, *P* = 0.002) showed significantly higher BCMA mRNA expression level compared to healthy individuals (median = 0.0006, IQR = 0.01). Nonetheless, there was not statistically significant difference between stable and cAMR patients (Figure 7). Also, classified patients according to the years post-transplantation (supplementary figure 4A) as well as drug regimens (supplementary figure 4B) did not show significant differences in the mRNA expression of BCMA.

### 3.7. mRNA Expression of TACI

**Figure 8:**
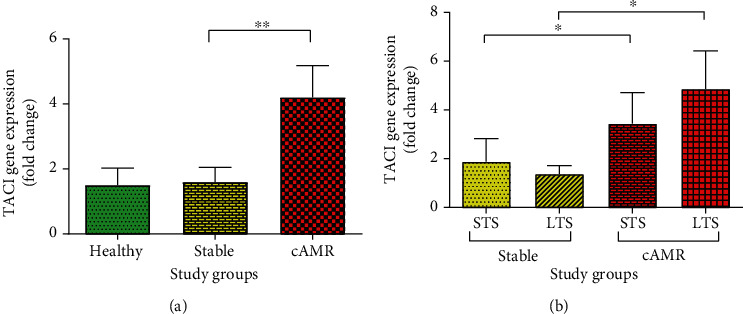
mRNA expression level of TACI in healthy individuals, patients with stable graft function, and cAMR patients (a). mRNA expression level of TACI in stable and cAMR patients with short-term survival (STS) and long-term survival (LTS) (b). Error bars represent SEM (^∗^*P* ≤ 0.05, ^∗∗^*P* ≤ 0.01).

Interestingly, it was observed that the mRNA expression of TACI had significant increase (*P* = 0.01) in the cAMR patients (median = 2.37, IQR = 4.66) compared to the stable patients (median 0.46, IQR = 1.84) (Figure 8(a)). Moreover, the stratification of patients by the years after transplantation showed that TACI mRNA expression level in the STS cAMR patients(median = 2.42, IQR = 2.57) was significantly (*P* = 0.029) higher than STS stable patients (median = 0.45, IQR = 1.83). The LTS cAMR patients (median = 2.11, IQR = 5.16) also showed significant (*P* = 0.04) overexpression of TACI mRNA expression level compare to LTS stable patients (median = 0.39, IQR = 1.90) (Figure 8(b)), but drug regimens had no significant effect on the mRNA expression of TACI in stable and cAMR patients (supplementary figure 5).

### 3.8. Correlation Analysis of BAFF

**Figure 9:**
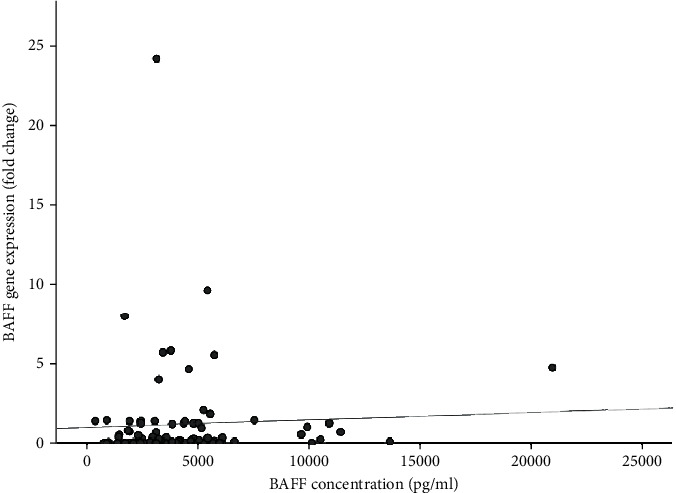
Correlation between serum and mRNA expression levels of BAFF.

The correlation of BAFF serum level was investigated with urea, creatinine, eGFR, years post-transplantation, age, and body mass index (BMI). It was observed that the BAFF serum level had a significant positive correlation (*r* = 0.26, *P* = 0.01) with blood urea level of patients. On the other hand, the BAFF mRNA expression did not have any correlation with urea, creatinine, eGFR, and BMI. However, we detected that the BAFF mRNA expression was positively correlated significantly with age (*r* = 0.27, *P* = 0.01) and years after transplantation (*r* = 0.36, *P* = 0.002) (Table 5). Moreover, our analysis showed that there was not any significant correlation between serum level and mRNA expression of BAFF (Figure 9).

### 3.9. Correlation Analysis of TACI mRNA Expression

A significant negative correlation (*r* = −0.22, *P* = 0.03) and a significant positive correlation (*r* = 0.28, *P* = 0.008) were observed between mRNA expression of TACI with eGFR and blood urea level, respectively. However, no correlation was detected between TACI mRNA expression level and creatinine, years post-transplantation, age, and BMI (Table 5).

### 3.10. BAFF and Its Receptors in Transplanted Patients according to the Donor Graft Sources

**Figure 10:**
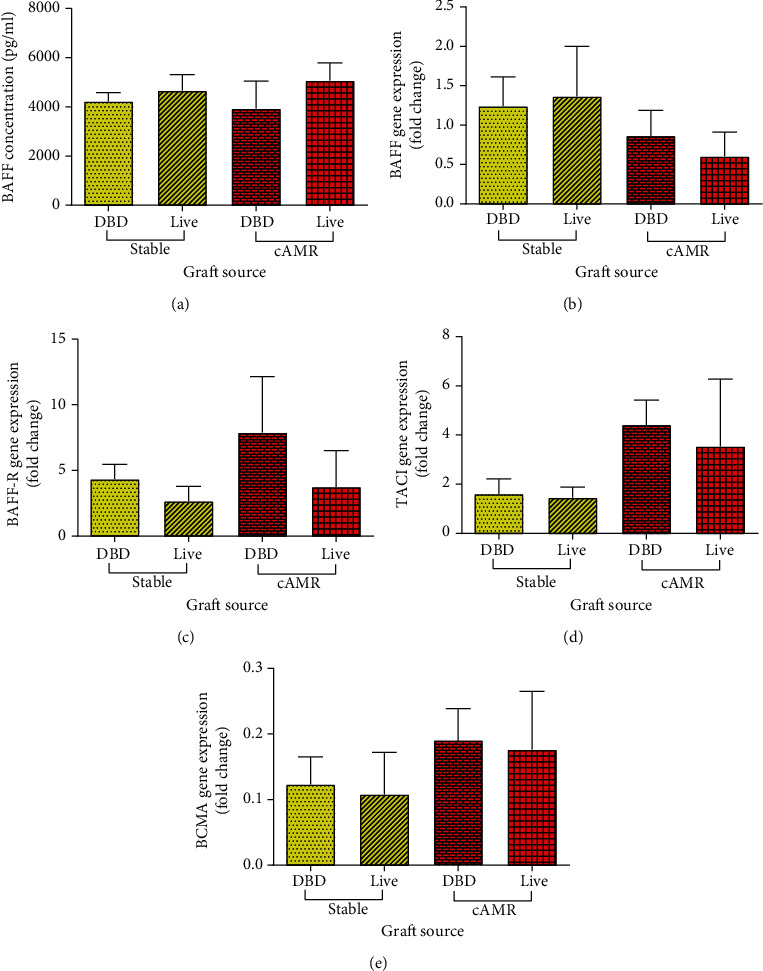
Comparing BAFF serum level (a), mRNA expression of BAFF (b), BAFF-R (c), TACI (d), and BCMA (e) between stable transplanted patients which received their graft from living or deceased donors and also cAMR patients which received their graft from living or deceased ones. Error bars represent SEM.

We stratified the patients based on the source of the donors, whether it was living or deceased (DBD, donation after brain death). The BAFF serum level and the mRNA expression of BAFF, BAFF-R, TACI, and BCMA were compared between stable transplanted patients with living or deceased graft source and also cAMR patients which received their graft from living or deceased ones. The results showed that there was not statically significant difference in BAFF serum level (Figure 10(a)) and the mRNA expression level of BAFF (Figure 10(b)), BAFF-R (Figure 10(c)), TACI (Figure 10(d)), and BCMA (Figure 10(e)) between living and deceased donor graft.

## 4. Discussion

A study on cardiac allograft mice indicated that mice with deletion of BAFF cytokine gene or mutant BAFF-R (BAFF-R^−/−^) have a prolonged graft survival compared to wild type mice [18]. This observation suggested that the absence of BAFF cytokine or its receptor may improve graft survival. On the other hand, it has been shown that patients whose peripheral B cells are depleted can still produce antibodies, and this antibody production was attributed to signals coming from BAFF. It has been demonstrated that BAFF stimulates TLO's B cells to produce antibody in situ [19]. Therefore, as the BAFF and its receptors seem to be involved in graft rejection, we designed this study to investigate the role of this cytokine and its receptors in the kidney transplant outcome.

In this study, the results showed a significant decrease in the number and percentage of lymphocytes in transplanted patients compared to healthy individuals, which seems to be a consequence of immunosuppressive therapy. Also, our results showed that the number and percentage of B cells in transplant patients have declined which is similar to some other studies in which a decline was shown in the absolute number of B cells in all transplanted patients compared to healthy subjects [20, 21]. We showed that BAFF serum levels in both cAMR patients and stable patients were significantly higher than healthy subjects, which is well-supported by other studies [21–23]. Xu et al. demonstrated that renal transplant patients had higher serum BAFF levels compared to healthy subjects [22]. Additionally, it has been shown that soluble BAFF in pediatric kidney transplant patients was higher than in healthy children [21]. Moreover, other investigators measured the membrane-bound form of BAFF by flow-cytometry and indicated that membrane-bound BAFF in patients with abnormal renal function is higher than healthy volunteers [23]. Our experiments also indicated that the transcript level of BAFF was enhanced in both stable and cAMR patients compared to healthy individuals. Taken together, despite lower B cell count in transplanted patients, their BAFF level is increased. According to previous studies, serum BAFF levels increase in autoimmune patients with B cell depletion after their immunosuppressive therapy [24–26]. Also, it has been indicated that BAFF level can be increased (more than 5000 pg/ml), in response to B cell depletion [27]. In addition, patients with chronic graft-versus-host disease (cGVHD) showed an increase in their BAFF level after depleting their B cells by rituximab [28]. Thaunat et al. reported that chronic kidney rejection patients with rituximab therapy and complete depletion of B cells in peripheral blood continued to produce antibodies, despite the absence of B cells in blood circulation [19]. Their further studies demonstrated that TLOs which include B cells were formed within cAMR renal allografts and had the ability to produce alloantibodies continuously [29, 30]. Also, their results displayed an increase in serum and gene expression of BAFF in B cell depleted cAMR patients, and they demonstrated that the signals which came from BAFF were inducing antibody production in TLOs and allowed in situ B cells to escape from apoptosis by rituximab [19]. Overall, this may be one of the reasons that our transplanted patients showed more BAFF in spite of peripheral B cell reduction. On the other hand, it has been shown that BAFF enhances B cells' chemotactic response to CXCL13 [31]. And it has been demonstrated that the high expression of CXCL13 and its receptor (CXCR5) in the transplanted allograft resulted in homing of CXCR5^+^ B cells to it [32]. Totally, it seems that there is a positive feedback between BAFF and intragraft B cells; it means that the more BAFF, the more B cells homing, and also, the more intragraft B cells, the more BAFF production, and this cycle can be repeated. There are some anti-BAFF drugs like atacicept with the ability to reduce BAFF levels which have been used for treating autoimmune diseases like systemic lupus erythematosus (SLE) [33]. It has been shown that atacicept has inhibited the early formation of DSAs and AMR development in nonhuman primates [34]; therefore, more investigations are required for assessment of the atacicept effect on cAMR patients.

In the next step of our study, graft rejection patients were compared with stable graft patients for understanding whether BAFF is involved in the determination of kidney allograft function or not. Different studies have shown conflicting results with respect to comparing BAFF in patients with abnormal kidney function and stable graft patients. Moreover, few studies have investigated BAFF and its receptors in cAMR patients. We could not find any difference in the serum level of BAFF between cAMR and stable patients. Also, some studies reported the same results, in which the soluble BAFF level is not associated with allograft rejection [35–38]. In line with our results, Koscielska-Kasprzak et al. investigated the long-term kidney transplant recipients (stable = 44 and chronic allograft dysfunction = 22) and reported that serum level of BAFF could not discriminate chronic allograft dysfunction patients from stable patients [35]. Besides, measuring soluble BAFF levels in 115 renal transplant patients, who undergone biopsy due to creatinine raise, showed that post-transplant soluble BAFF levels did not have any effect on the appearance of donor-specific antibodies, biopsy findings, allograft rejection, and other allograft outcomes [36]. Additionally, 101 patients who were waiting for transplantation were monitored up to one year post-transplantation for the incidence of AMR, and results showed that there was no association between pretransplantation soluble BAFF and AMR frequency [37]. Also, an investigation of patients undergoing antibody compatible transplantation showed no association between soluble BAFF level and risk of AMR development [38].

Xu et al. study included 69 renal transplant recipients, 13 healthy volunteers, and 18 patients with renal abnormal function and classified the patients according to post-transplantation follow-up duration into three groups: less than one year, between one to four years, and equal or more than five years. Their results showed that both mRNA level and membrane-bound BAFF expression were increased with the time after transplantation [39]. Likewise, in our study, BAFF transcript showed a significant positive correlation with years post-transplantation (Table 5); as well, in both stable and cAMR patients, long-term survival (LTS) patients showed more BAFF transcript compared to short-term survival (STS) patients (Figure 6(b)). Totally, it shows that although BAFF may not differentiate stable and cAMR patients, it increases over the years post-transplantation. So it has the potential to be studied more to understand whether this high long-lasted BAFF affects the patient's outcome or not.

On the other hand, Xu et al. showed that BAFF in AMR patients was higher than the control group and indicated that BAFF is associated with AMR in the transplanted patients [40]. Notably, in this study, BAFF was detected by immunohistochemical staining in the renal biopsies, while we applied the ELISA method for measuring soluble BAFF levels in cAMR transplant recipients. This may suggest that the soluble form of BAFF cannot distinguish patients with graft rejection and theirs with stable graft, rather measuring it in biopsy samples can be useful. Another suggestion is that measuring BAFF may detect AMR, not cAMR patients. On the other hand, Pongpirul et al. showed that AMR can be predicted by soluble BAFF monitoring [8]. However, there are some differences in their study design that may explain the discrepancy of their findings compare to us. They enrolled 68 rejection free transplanted patients in their study and measured their soluble BAFF and classified the patients by their BAFF concentration to high or low BAFF level groups. The patients were investigated for AMR in 6 months post-transplantation, and it was indicated that AMR was more probable in the high BAFF level group. Also, they measured BAFF before recognizing AMR, while we detected the BAFF level after biopsy-proven cAMR. As well, experiments on the pretransplant soluble BAFF level indicated that BAFF had a negative effect on graft survival, and high BAFF level can be a risk factor for AMR [41]. This implies that the time for evaluation of BAFF can impress the results.

In addition, our results showed that there was no significant correlation between serum level and mRNA expression of BAFF. This can be due to the difference in their sources, BAFF protein level was measured in serum, while PBMCs were used for its gene expression evaluation. On the other hand, as it mentioned previously, BAFF exists in both membrane and soluble form, and the mRNA expression is attributed to both of them, while ELISA just measures the soluble form.

Additionally, according to higher BAFF concentration in cAMR patients who received CsA compared to patients who received Tac (Figure 5(b)), it seems that Tac may be a better immunosuppressive drug in comparison to CsA. Likewise, some studies have shown that Tac is better than CsA to use in renal transplant patients [42, 43]. Penninga et al. designed a systematic review and meta-analyses study and indicated that Tac seems to be superior to CsA as an immunosuppressive drug [44].

Few studies have investigated the expression of BAFF receptors (BAFF-R, BCMA, and TACI) in kidney transplantation, and the results of these studies are conflicting. Our results showed that TACI is the only receptor that was increased in cAMR patients compared to stable recipients. This observation may stem from the fact that TACI is a common receptor for both BAFF and APRIL cytokines and binds to BAFF with high affinity. Also, the TACI signaling pathway is different from two other receptors. TACI has an extra ligand-binding site, which can be the cause of its different results compared to BAFF-R and BCMA. Our results showed that the TACI expression was increased in cAMR patients compared to stable patients, and this increased TACI remains high over time. When we investigated the TACI expression changes in short- and long-term survival patients, the same results were shown for both time points. It means that same as STS cAMR patients which had more TACI compared to STS stable patients, LTS cAMR patients showed more TACI in comparison to LTS stable patients too (Figure 8(b)). Also, we found that BCMA in cAMR and stable patients is higher than in healthy individuals (Figure 7). Minz et al. evaluated the mRNA expression level of BAFF-R, TACI, and BCAM in 36 stable and 40 rejected patients, before kidney transplantation and 1, 3, 6, and 12 month posttransplantations and at the time of rejection. They reported that the expression of BAFF receptors showed no significant difference in nonrejected patients at the previously mentioned time points. But rejected patients showed a significant increase in their receptor gene expression level [45]. Their study has before-after comparing design, and they compared each group with their pretransplant phase, while we compared the expression of the receptors between stable and cAMR groups after transplantation.

By investigating 143 transplanted patients at three time points include the time of renal dysfunction, the time of anti-HLA antibody appearance, and the time of DSA development, Thibault-Espitia et al. reported no correlation between serum and mRNA expression levels of BAFF, same as our results. But this study reported that both serum and mRNA expression levels of BAFF correlated negatively with TACI and BAFF-R transcripts, while they did not have any correlation with BCMA. In addition, they indicated that patients with upregulated BAFF-R expression were more prone to developing renal dysfunction, and also, patients with low BAFF transcripts and high soluble BAFF levels had a higher risk to develop DSAs [23]. Again, there is a big difference in our study design that may be the underlying reasons for incongruous results. Thibault-Espitia et al. considered 3 different time points for all transplant recipients, while we measured BAFF receptors in three distinct groups (stable, cAMR, and healthy). Overall, it seems that according to the controversial results in different studies, more multicentral cohort studies are needed to find out the importance of BAFF receptors in kidney transplantation.

## 5. Conclusion

Overall, in spite of lower B cell count in transplanted patients because of lymphocyte depleting immunosuppressive drugs, their BAFF level is increased. Both soluble protein and mRNA transcript of BAFF increased in transplant recipients; as well, BAFF transcripts had a positive correlation with years post-transplantation and increased over the years after transplantation in both stable and cAMR patients. However, BAFF neither at the serum level nor at the mRNA transcript level cannot be a good biomarker for prediction of cAMR. In the case of receptors, TACI as a common receptor for both APRIL and BAFF is more important compared to other receptors, because it has a higher expression level in cAMR patients compare to stable patients, and it may distinguish cAMR and stable patients, even years after transplantation, because both STS and LTS cAMR patients have increased TACI compared to STS and LTS stable patients. Nonetheless, further studies are still required to disclose the clear involvement of TACI in the context of kidney transplantation.

## Figures and Tables

**Table 1:** Demographic data of study groups.

Variables	Study groups			*P* value
	Healthy individuals	Stable patients	cAMR patients	
Number	8	40	40	—

Gender				0.810
Male	4 (50%)	27 (67.5%)	26 (65%)	
Female	4 (50%)	13 (32.5%)	14 (35%)	

Age (years)	27.75 (3.10)	42.82 (14.21)	44.02 (15.14)	0.010
	26.50 [26; 28]	41.50 [30.75; 49.00]	39.00 [33.00; 57.50]	

Weight (kg)	67.37 (12.02)	72.65 (10.58)	73.13 (17.60)	0.546
	65.50 [60.75; 74.25]	73 [65.25; 82.00]	73.25 [59.87; 83.12]	

Height (m)	1.71 (0.07)	1.69 (0.10)	1.68 (0.11)	0.566
	1.70 [1.65; 1.76]	1.70 [1.61; 1.76]	1.70 [1.60; 1.75]	

BMI^1^ (kg; m^2^)	23.03 (4.21)	25.46 (3.56)	25.69 (5.1)	0.254
	21.96 [20.17; 24.79]	25.59 [22.84; 27.26]	24.74 [22.39; 30.07]	

Donor type				0.120
Living donor	—	13 (32.5%)	8 (20%)	—
Deceased donor	—	27 (67.5%)	32 (80%)	—

Months post TX^2^	—	69.59 (42.41)	72.55 (61.06)	0.310
	—	60.00 [36.0; 84.00]	60.00 [24.00; 120.00]	

Drug regimen				
Pred^3^, MMF^4^, CsA^5^	—	17 (42.5%)	22 (55%)	—
Pred, MMF, Tac^6^	—	23 (57.5%)	18 (45%)	—

Blood group				0.095
O	4 (50%)	7 (17.5%)	10 (25%)	—
A	1 (12.5%)	11 (27.5%)	8 (20%)	—
B	2 (25%)	14 (35%)	6 (15%)	—
AB	1 (12.5%)	3 (7.5%)	10 (25%)	—
Unknown	0 (0%)	5 (12.5%)	6 (15%)	—

Underlying disease				
Diabetes mellitus	—	9 (22.5%)	15 (37.5%)	0.010
Hypertension	—	14 (35%)	32 (90%)	0.011
Hypothyroidism	—	3 (7.5%)	1 (2.5%)	0.420
Cardiac disease	—	9 (22.5%)	6 (15%)	0.091

Values were expressed as mean (standard deviation), median [Q1; Q3], or number (percentage). ^1^BMI. ^2^Transplantation. ^3^Prednisolone. ^4^Mycophenolate mofetil. ^5^Cyclosporine A. ^6^Tacrolimus.

**Table 2:** Baseline characteristics of study groups.

Variables	Study groups			*P* value
	Healthy individuals	Stable patients	cAMR patients	
eGFR^1^ (ml/min/1.73 m^2^)	107.37 (9.50)	74.76 (30.03)	49.49 (33.01)	<0.001
	109.00 [98.50; 113.50]	79.71 [56.01; 97.70]	39.72 [18.53; 82.42]	

Creatinine (mg/dl)	0.81 (0.23)	1.23 (0.68)	3.30 (3.70)	<0.001
	0.85 [0.60; 0.92]	1.24 [1.09; 2.01]	3.16 [2.01; 5.9]	

Urea (mg/dl)	26.75 (8.75)	41 (26)	88 (75.5)	<0.001
	27.00 [19.50; 31.75]	44.00 [31.00; 58.50]	86.5 [44.00; 123.50]	

BUN^2^	17.12 (6.49)	25.98 (19.25)	44.76 (24.22)	<0.001
	18.50 [13.50; 21.25]s	19.15 [14.01; 26.63]	41.58 [28.50; 58.41]c	

TG^3^ (mg/dl)	92.87 (33.15)	161.79 (107.72)	165.51 (73.97)	0.192
	94.50 [76.50; 110.25]	140 [103.50; 175]	147.00 [120.00; 203.00]	

Cholesterol (mg/dl)	142.5 (42.59)	149.42 (35.32)	153.79 (56.69)	0.919
	125.00 [108.50; 189.50]	141.00 [123.00; 172.00]	143.00 [123.00; 186.50]	

HDL^4^ (mg/dl)	48.37 (9.78)	45.24 (20.08)	48.82 (23.93)	0.671
	47.50 [43.75; 56.75]	41.00 [31.00; 52.00]	45.00 [37.00; 55.50]	

LDL^5^ (mg/dl)	105.25 (20.28)	89.38 (39.83)	76.90 (26.12)	0.624
	110.00 [99.75; 115.50]	80.00 [6.50; 105.50]	75.00 [63.00; 98.00]	

WBC^6^ (10^3^/*μ*l)	6.25 (1.28)	6.54 (2.35)	7.09 (5.66)	0.756
	6.25 [5.45; 7.12]	6.10 [4.84; 8.35]	6.17 [4.92; 8.31]	

RBC^7^ (10^6^/*μ*l)	5.10 (0.77)	4.25 (0.97)	3.27 (1.16)	0.003
	5.00 [4.80; 5.80]	4.10 [3.60; 4.81]	3.31 [2.94; 4.00]	

Hb^8^ (g/dl)	12.53 (1.18)	12.62 (2.85)	9.84 (1.77)	<0.001
	12.2 [11.8; 13.35]	12.80 [10.95; 14.55]	9.60 [8.60; 11.20]	

Platelet (103/*μ*l)	295.00 (109.80)	201.35 (77.29)	168.78 (100.69)	0.996
	280.00 [212.5; 387.50]	198.00 [149.0; 249.5]	178.00 [121.50; 221.00]	

Lymphocyte (%)	36.41 (5.62)	21.20 (9.32)	22.32 (9.70)	<0.001
	[32.00; 38.00]	19.00 [14.00; 26.50]	23.52 [16.00; 29.00]	

FBS^9^ (mg/dl)	106.12 (7.88)	113.94 (54.84)	119.57 (72.89)	0.882
	104 [99.75; 111.00]	97.00 [88.00; 104.50]	97.00 [86.00; 115.25]	

Calcium (mg/dl)	9.27 (0.83)	11.66 (15.65)	10.34 (10.67)	0.051
	9.10 [8.87; 10.00]	9.25 [8.70; 9.67]	8.75 [8.27; 9.22]	

Phosphor (mg/dl)	3.83 (0.76)	3.75 (1.20)	6.36 (8.18)	<0.001
	3.70 [3.30; 4.50]	3.60 [3.00; 4.10]	4.85 [3.87; 6.00]	

Sodium (mEq/l)	140.00 (3.92)	103.44 (3.07)	137.85 (5.33)	0.016
	139.5 [136.75; 144.00]	141.00 [138.85; 142.00]	138.00 [136.00; 141.00]	

Potassium (mEq/l)	5.20 (1.11)	4.39 (0.93)	4.68 (0.98)	0.062
	5.30 [4.90; 6.00]	4.15 [3.92; 4.56]	4.60 [4.17; 5.04]	

AST^10^ (U/l)	26.75 (10.67)	21.30 (6.75)	18.97 (10.80)	0.027
	29.00 [17.25; 33.75]	21.00 [16.00; 27.00]	15.50 [11.75; 24.00]	

ALT^11^ (U/l)	32.25 (14.28)	31.05 (27.05)	26.15 (30.75)	0.034
	34.5 [20.50; 42.00]	26.00 [16.00; 32.00]	15.50 [12.75; 24.00]	

Values were expressed as mean (standard deviation) and median [Q1; Q3]. ^1^Estimated glomerular filtration rate. ^2^Blood urea nitrogen. ^3^Triglyceride. ^4^High density lipoproteins. ^5^Low density lipoproteins. ^6^White blood cells. ^7^Red blood cell. ^8^Hemoglobin. ^9^Fasting blood sugar. ^10^Aspartate aminotransferase. ^11^Alanine aminotransferase.

**Table 3:** Estimated GFR formula.

	SCr^1^ ≤ 0.7 mg/dl	SCr > 0.7 mg/dl
Nonblack female	144 ^∗^ (SCr/0.7)-0.329 ^∗^ (0.993) age	144 ^∗^ (SCr/0.7)-1.209 ^∗^ (0.993) age
Nonblack male	141 ^∗^ (SCr/0.9)-0.411 ^∗^ (0.993) age	141 ^∗^ (SCr/0.9)-1.209 ^∗^ (0.993) age

^1^Serum creatinine.

**Table 4:** Primers.

Primers	Forward (5′ to 3′)	Reverse (5′ to 3′)
BAFF	GGCCCCAACCTTCAAAGTTC	GCGTGACTGCTCCCTTTCTG
BAFF-R	CCCTGGACAAGGTCATCATT	TCTTGGTGGTCACCAGTTCA
BCMA	GCAGTGCTCCCAAAATGAAT	GTCCCAAACAGGTCCAGAGA
TACI	AGTGAACCTTCCACCAGAGC	CTCTTCTTGAGGAAGCAGGC

**Table 5:** Correlation of BAFF and its receptors with some demographic characteristics.

Parameter	Correlation coefficient	*P* value
Soluble BAFF		
Urea	0.26	0.01
Creatinine	0.2	0.57
eGFR^1^	-0.12	0.24
Years after TX^2^	0.02	0.86
Age	0.06	0.58
BMI^3^	0.04	0.69
BAFF gene expression		
Urea	-0.12	0.34
Creatinine	0.1	0.39
eGFR	-0.06	0.61
Years after TX	0.36	0.002
Age	0.27	0.01
BMI	0.06	0.59
BAFF-R gene expression		
Urea	0.14	0.18
Creatinine	0.06	0.57
eGFR	-0.015	0.15
Years after TX	0.13	0.25
Age	0.15	0.16
BMI	0.16	0.13
TACI gene expression		
Urea	0.284	0.008
Creatinine	0.001	0.99
eGFR	-0.22	0.03
Years after TX	-0.02	0.83
Age	0.17	0.1
BMI	-0.03	0.75
BCMA gene expression		
Urea	0.03	0.47
Creatinine	0.08	044
eGFR	-0.16	0.12
Years after TX	0.02	0.85
Age	0.13	0.2
BMI	0.14	0.18

^1^Estimated glomerular filtration rate. ^2^Transplantation. ^3^Body mass index.

## Data Availability

The data used to support the findings of this study are available from the corresponding author upon request.

## Abbreviations

cAMR: Chronic antibody-mediated rejection
BAFF-R: BAFF receptor
BCMA: B cell maturation antigen
TACI: Transmembrane activator and cyclophilin ligand interactor
DSA: Donor specific antibody
HLA: Human leukocyte antigen
ADCC: Antibody-dependent cellular cytotoxicity
TLO: Tertiary lymphoid organ
CRD: Cysteine-rich domain
TRAF: TNF receptor associated factor
NF-*κ*B: Nuclear factor-*κ*B
NIK: NF-*κ*B inducing kinase
IKK1: Inhibitor of *κ*B kinase 1
AP-1: Activator protein 1
NFAT: Nuclear factor of activated T-cells
eGFR: Estimated glomerular filtration rate
MMF: Mycophenolate mofetil
STS: Short-term survival
LTS: Long-term survival.

## Supplementary Materials

Supplementary Materials Supplementary figure 1: BAFF concentration in stable and cAMR patients with short-term survival (STS) and long-term survival (LTS). Supplementary figure 2: BAFF gene expression in transplanted patients with different immunosuppressive drug, tacrolimus (Tac), or cyclosporine A (CsA). Supplementary figure 3: mRNA expression level of BAFF-R in patients with stable graft function, cAMR patients, and healthy individuals (a). mRNA expression level of BAFF-R in stable and cAMR patients with short-term survival (STS) and long-term survival (LTS) (b) and in stable and cAMR patients with different immunosuppressive drug, tacrolimus (Tac), or cyclosporine A (CsA) (c). Supplementary figure 4: mRNA expression level of BCMA in stable and cAMR patients with short-term survival (STS) and long-term survival (LTS) (a) and in stable and cAMR patients with different immunosuppressive drug, tacrolimus (Tac), or cyclosporine A (CsA) (b). Supplementary figure 5: TACI gene expression in transplanted patients with different immunosuppressive drug, tacrolimus (Tac), or cyclosporine A (CsA).
