# Supplementary material for: Investigating the Role of BAFF and Its Receptors in Renal Transplant Recipients with Chronic Antibody-Mediated Rejection
Source: J Immunol Res. 2021 Mar 6;2021:6654992. doi: 10.1155/2021/6654992 (PMC7959970; doi:10.1155/2021/6654992)
Supplement: Supplementary Materials — Supplementary figure 1: BAFF concentration in stable and cAMR patients with short-term survival (STS) and long-term survival (LTS). Supplementary figure 2: BAFF gene expression in transplanted patients with different immunosuppressive drug, tacrolimus (Tac), or cyclosporine A (CsA). Supplementary figure 3: mRNA expression level of BAFF-R in patients with stable graft function, cAMR patients, and healthy individuals (a). mRNA expression level of BAFF-R in stable and cAMR patients with short-term survival (STS) and long-term survival (LTS) (b) and in stable and cAMR patients with different immunosuppressive drug, tacrolimus (Tac), or cyclosporine A (CsA) (c). Supplementary figure 4: mRNA expression level of BCMA in stable and cAMR patients with short-term survival (STS) and long-term survival (LTS) (a) and in stable and cAMR patients with different immunosuppressive drug, tacrolimus (Tac), or cyclosporine A (CsA) (b). Supplementary figure 5: TACI gene expression in transplanted patients with different immunosuppressive drug, tacrolimus (Tac), or cyclosporine A (CsA). [file 6654992.f1.docx]

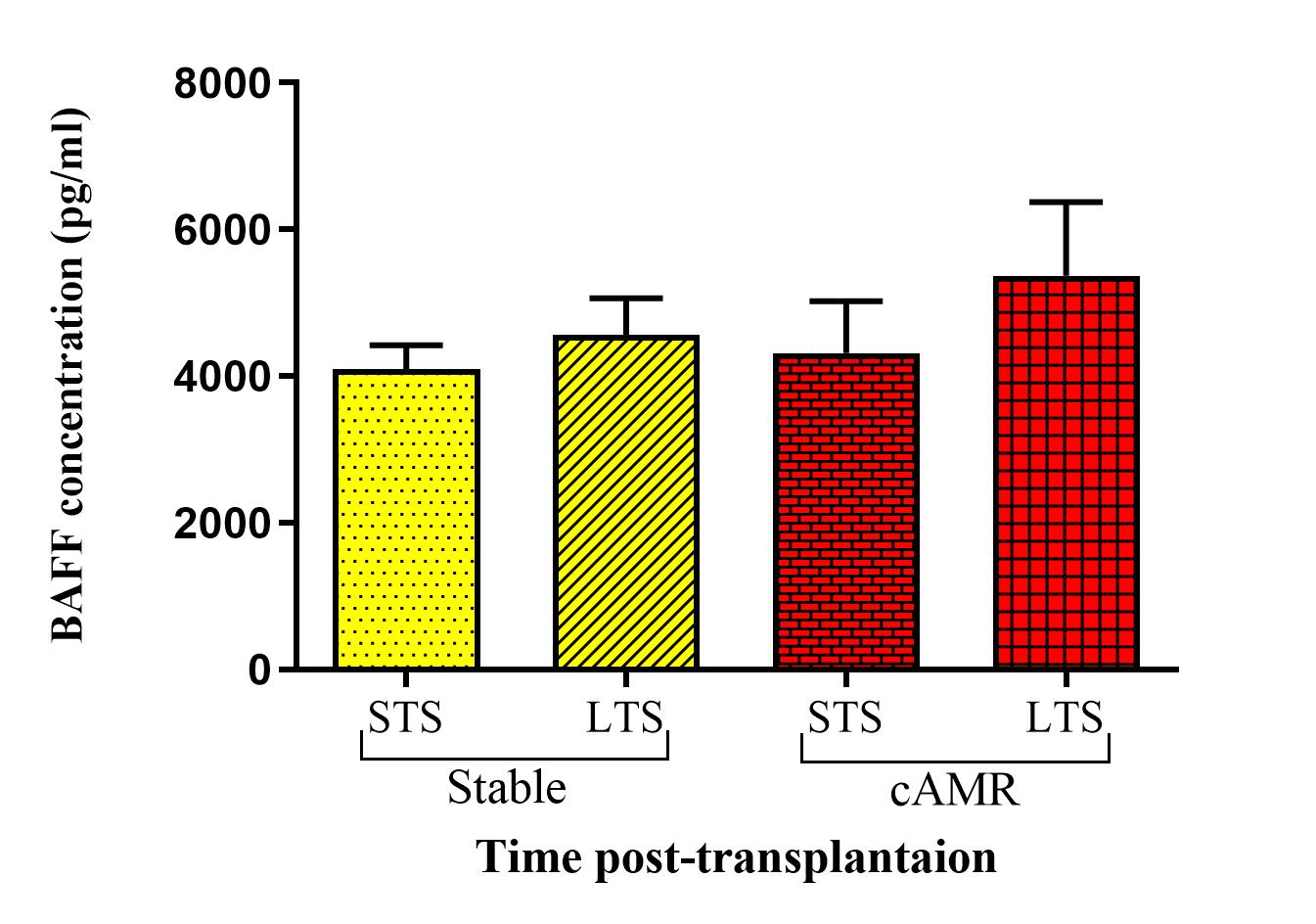


**Supplementary figure 1**. BAFF concentration in stable and cAMR patients with short-term survival (STS) and long-term survival (LTS). Error bars represent SEM.


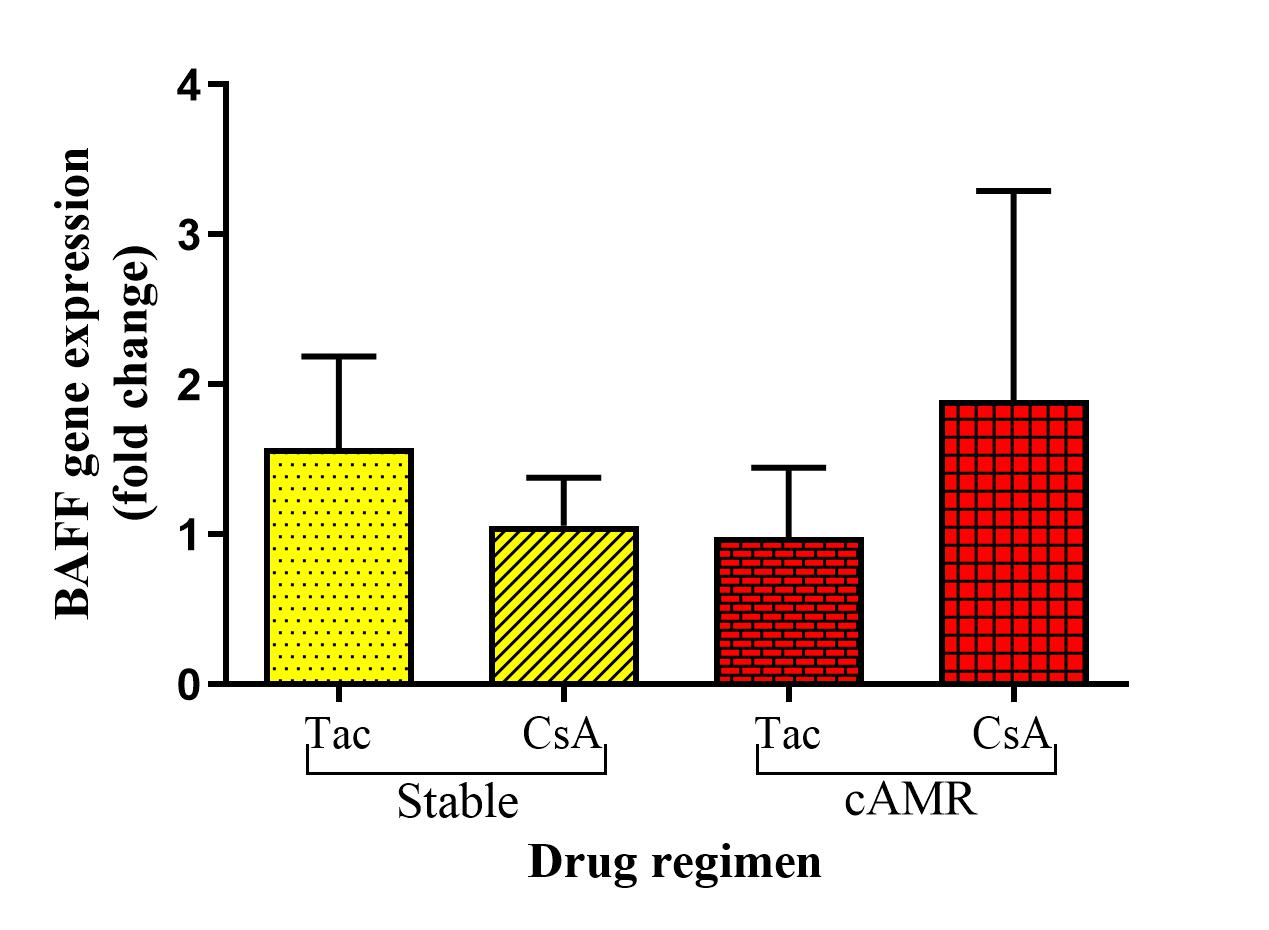


**Supplementary figure 2**. BAFF gene expression in transplanted patients with different immunosuppressive drug, tacrolimus (Tac) or cyclosporine A (CsA). Error bars represent SEM.


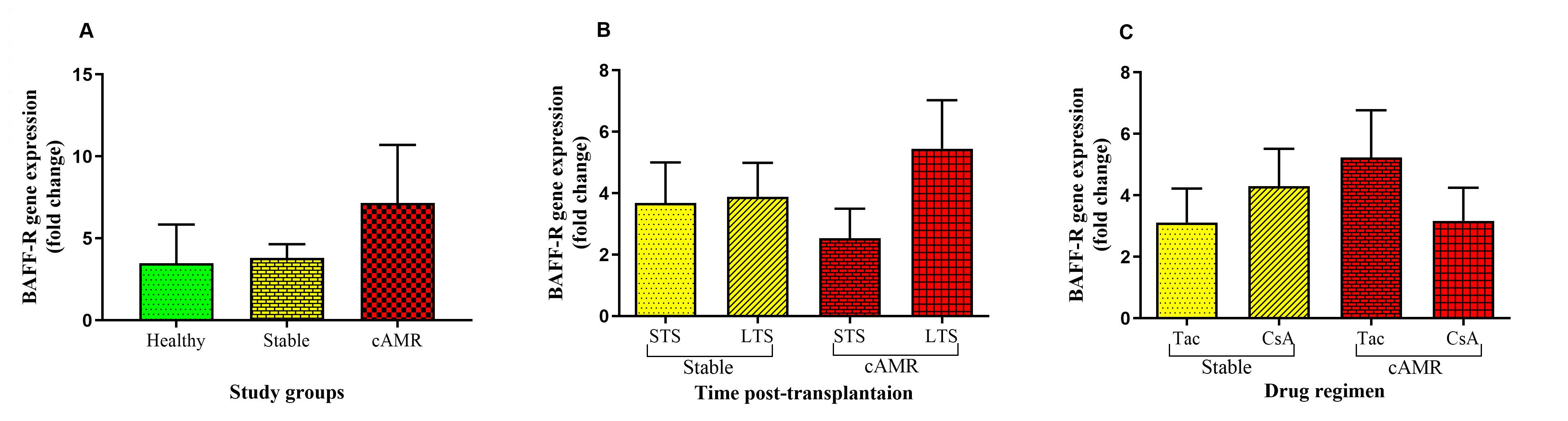


**Supplementary figure 3**. mRNA expression level of BAFF-R in patients with stable graft function, cAMR patients, and healthy individuals (A). mRNA expression level of BAFF-R in stable and cAMR patients with short-term survival (STS) and long-term survival (LTS) (B) and in Stable and cAMR patients with different immunosuppressive drug, tacrolimus (Tac) or cyclosporine A (CsA) (C). Error bars represent SEM.


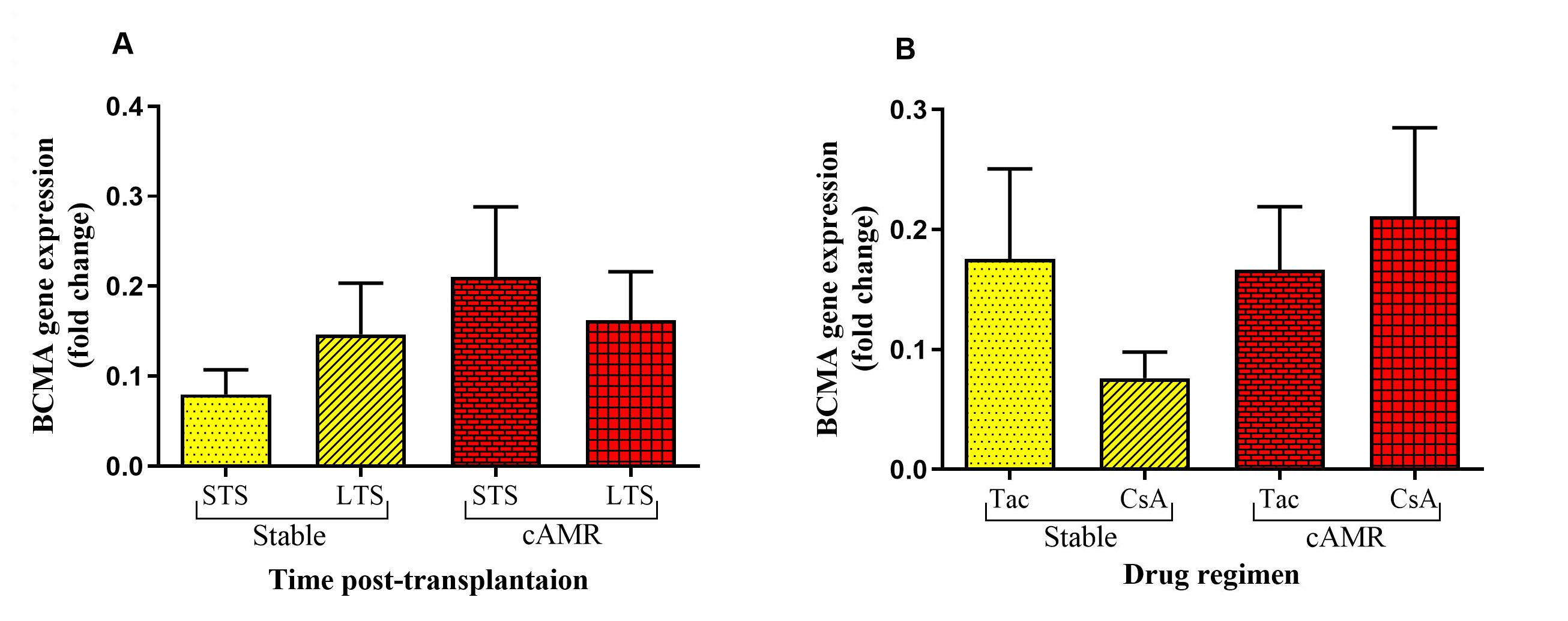


**Supplementary figure 4**. mRNA expression level of BCMA in stable and cAMR patients with short-term survival (STS) and long-term survival (LTS) (A) and in Stable and cAMR patients with different immunosuppressive drug, tacrolimus (Tac) or cyclosporine A (CsA) (B). Error bars represent SEM.


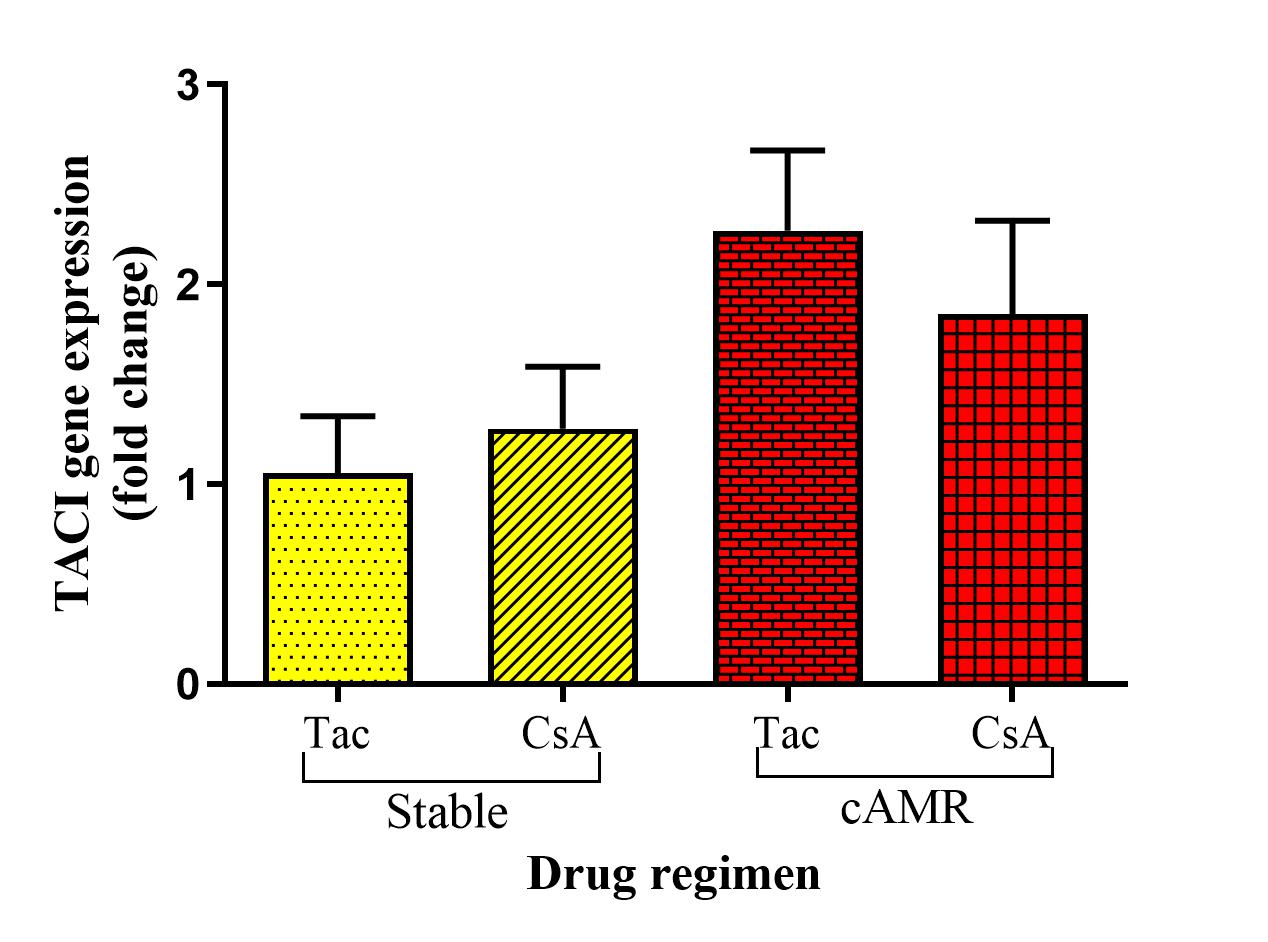


**Supplementary figure 5**. TACI gene expression in transplanted patients with different immunosuppressive drug, tacrolimus (Tac) or cyclosporine A (CsA). Error bars represent SEM.
